# AI Versus Human-Delivered Online Cognitive Behavioral Therapy for Anxiety Symptoms in Young Adults: A Randomized Controlled Trial

**DOI:** 10.3390/healthcare14101325

**Published:** 2026-05-13

**Authors:** Weihao Huang, Yiyang Wu, Yujin Shen, Haoran Song, Chen Ye, Ruoyu Lin, You Wang, Xueling Yang

**Affiliations:** 1 Department of Psychology, School of Public Health, Southern Medical University, Guangzhou 510515, China; 19925653004@163.com (W.H.); 13113346151@163.com (Y.W.); shr13360178370@163.com (H.S.); 13126319897@163.com (C.Y.); 13068622614@163.com (R.L.); 2 Department of Psychiatry, Brain Hospital Affiliated to Guangzhou Medical University (Guangzhou Huiai Hospital), Guangzhou 510370, China; 18142816217@163.com; 3 Department of Psychiatry, Zhujiang Hospital, Southern Medical University, Guangzhou 510515, China

**Keywords:** AI, anxiety, cognitive behavioral therapy, intervention, qualitative analysis

## Abstract

Objective: This study aimed to compare the effectiveness of online cognitive behavioral therapy (CBT) delivered by an AI chatbot versus human peer counselors (participants were told it was AI) in reducing anxiety symptoms in young adults. Methods: Ninety young adults with mild-to-severe anxiety were randomized to a 4-week intervention of AI-CBT (*n* = 30), peer-counselor-CBT (*n* = 30), or a no-intervention control (*n* = 30). The primary outcome, anxiety, was assessed at baseline, mid-point, and post-intervention. Secondary outcomes (the self-efficacy for exercise, sleep quality), psychotherapy benefit, and qualitative user experiences were also evaluated. Results: Both AI and human-delivered interventions led to significant within-group reductions in anxiety (*p* < 0.05). However, in the primary intention-to-treat analysis, neither intervention demonstrated a statistically significant advantage over the no-intervention control group at post-intervention. A secondary per-protocol analysis suggested a benefit for the human-delivered intervention among study completers. Notably, participants in the AI group reported significantly lower perceived treatment benefit than the human group (*p* < 0.001). Qualitative analyses indicated that while AI was valued for accessibility and consistency, human intervention was perceived as more flexible in guidance, individualized, emotionally supportive, and conducive to deeper exploration. Conclusions: In this exploratory trial, both AI- and peer-counselor-CBT showed within-group promise, but the evidence does not support their efficacy over a no-intervention control. The AI’s limitations in providing flexible, emotionally supportive, and personalized interaction likely explain the efficacy gap observed between the two interventions. While AI may serve as a scalable support tool, claims of clinical efficacy require significant caution. These preliminary findings warrant replication in a prospectively registered confirmatory trial.

## 1. Introduction

Anxiety disorders, a prevalent mental health concern globally, have exerted a significant impact on individuals and society for many years. A 2021 systematic review published in *The Lancet* on the global epidemiology of mental health disorders revealed a concerning trend. Since the outbreak of the COVID-19 pandemic, the prevalence of anxiety disorders has risen to approximately 4.8%, equating to approximately 374 million people worldwide. This represents an alarming increase of about 25.6% compared to pre-pandemic levels [1]. Within this vast population of individuals grappling with anxiety, those in early adulthood and youth are particularly affected [2]. Anxiety disorders not only diminish patients’ quality of life, impacting sleep [3], physical activity [4], cognitive function [5], and interpersonal relationships [6], but also contribute to the deterioration of their physical health. Conditions like cardiovascular disease, gastrointestinal disorders, chronic pain, and migraines are often exacerbated by anxiety [7]. These consequences result in a substantial economic burden, including increased healthcare costs and decreased productivity. Furthermore, the pandemic hindered access to in-person mental health support from therapists, highlighting the need for advancements in current psychological treatment and intervention modalities.

Several psychotherapeutic approaches address anxiety disorders, including mindfulness-based interventions [8], acceptance and commitment therapy [9], psychodynamic therapy [10], emotion-focused therapy [11], and dialectical behavior therapy [12]. However, the efficacy of these therapies has not been consistently proved to be superior to that of Cognitive Behavioral Therapy (CBT). Indeed, clinical practice guidelines consistently recommend CBT as the first-line psychological treatment for anxiety disorders [13]. This recommendation is supported not only by its direct efficacy for anxiety symptoms but also because CBT techniques can effectively address mechanisms underlying common co-occurring issues: for instance, it can target the cognitive hyperarousal and worry contributing to sleep problems [14] and the avoidance behaviors or lack of confidence hindering physical activity [15]. This broader effectiveness provides theoretical support for our study’s hypotheses regarding improvements in sleep quality and exercise self-efficacy following CBT intervention. The core components of CBT encompass psychoeducation, cognitive restructuring, behavioral activation, and exposure techniques. Psychoeducation involves discussing the interplay between thoughts, feelings, and behaviors related to anxiety, helping patients understand their symptoms and maladaptive patterns. Cognitive restructuring focuses on identifying and challenging anxiety-related thoughts (e.g., catastrophizing) and teaching coping skills to reframe unhelpful thinking patterns (e.g., fear of uncertainty in generalized anxiety disorder, fear of negative evaluation in social anxiety disorder). Behavioral activation aims to increase engagement in rewarding activities. Exposure techniques, another key component, often involve guided, systematic confrontation with feared objects or situations in a safe manner to decrease avoidance and fear responses [16]. Despite its established efficacy, implementing CBT in real-world settings presents challenges. Research indicates that low perceived need for treatment is a leading cause for refusing treatment among individuals with DSM-IV disorders within the past 12 months, often accompanied by attitudinal barriers. This trend is more prevalent in mild and moderate cases compared to severe cases [17]. Moreover, the delivery of evidence-based therapies faces persistent obstacles due to an uneven distribution of trained providers, delays in treatment provision, and insufficient treatment capacity [18,19]. Innovative solutions are emerging to address these limitations. Internet-based psychological interventions, particularly internet-based cognitive behavioral therapy (iCBT), have gained recognition for their effectiveness, supported by robust evidence from systematic reviews and meta-analyses [20]. Unlike traditional CBT, iCBT empowers users to access treatment materials privately and conveniently, facilitating cost-effective, large-scale management and circumventing barriers inherent in conventional mental healthcare systems [21,22]. However, internet-based interventions still encounter challenges, such as relatively low adherence rates, issues with sustainability, and limitations in flexibility [23,24]. Addressing these shortcomings necessitates the integration of novel technologies.

In recent years, the rapid advancement of artificial intelligence (AI) has led to the emergence of AI-powered chatbots such as ChatGPT, AI Bing, Alexa, and Amazon Lex. These chatbots simulate conversational agents by processing users’ voice or text inputs to understand their meaning and generate meaningful responses or actions [25]. With the increasing ubiquity of smart mobile devices (smartphones, tablets, and computers), these chatbots have the potential to deliver accessible, autonomous, and engaging health-related information and services, holding great promise for technological innovation in intervention development [26]. Recent randomized controlled trials suggest that AI-powered chatbots can be effectively integrated into therapeutic interventions across a range of mental health concerns, including anxiety [27], depression [28], and substance abuse [29]. These positive findings are likely due to the distinct advantages of chatbots. For instance, a meta-analysis highlighted their flexibility in providing on-demand support, personalized interactions, and inherent sustainability, addressing limitations observed in traditional telehealth services [30]. While a growing body of literature explores the delivery of therapy through chatbots on smart mobile devices, demonstrating the effectiveness and distinct advantages of health behavior change interventions across diverse populations, research in this domain remains in its early stages. Rigorous randomized controlled trials are still needed to provide a nuanced understanding of the effects of AI-delivered interventions [26].

Despite the growing body of research on AI-powered chatbots for mental health interventions, key questions remain regarding their comparative effectiveness and underlying mechanisms. To address this gap, the present study employed two key methodological features. First, to disentangle technological effects from user expectancies, we utilized an innovative single-blind, deception-based design. Second, we adopted a mixed-methods approach to explore the nuanced differences in user experience. However, it is essential to note that this trial constitutes a post hoc, exploratory secondary analysis of a subsample from a larger pre-registered project. This fundamentally positions our objectives as hypothesis-generating. Acknowledging this context, we hypothesized that both AI-CBT and peer-counselor-delivered CBT would reduce anxiety symptoms, improve sleep quality, and enhance exercise self-efficacy. Furthermore, we anticipated that our qualitative analysis would illuminate distinct user experiences, thereby generating critical insights for the future refinement of AI-driven mental health interventions.

## 2. Methods

### 2.1. Study Design

**Figure 1:**
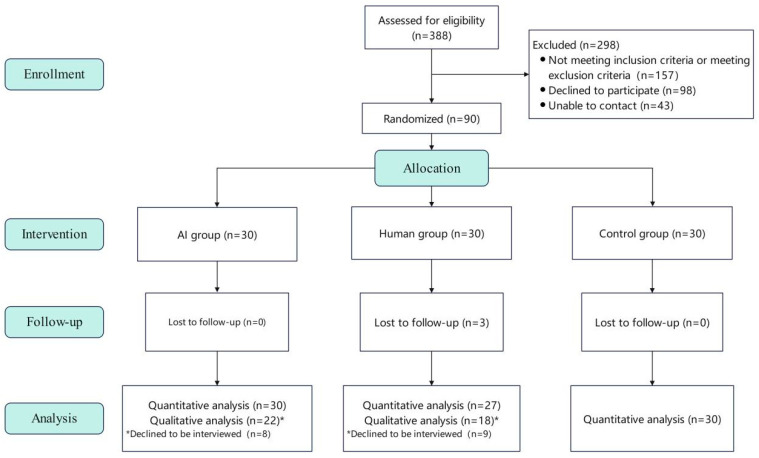
The flow of participants.

The design and reporting of this clinical trial adhere to the Consolidated Standards of Reporting Trials (CONSORT) 2010 statement [31] and its extension for artificial intelligence interventions (CONSORT-AI) [32]. This single-blind randomized controlled trial was conducted between August 2024 and October 2024, involving three parallel groups (see Figure 1): an AI intervention group (AI group), an online human intervention group (Human group), and a no-intervention Control group (Control group).

This study was registered with the Chinese Clinical Trial Registry (Registration Number: ChiCTR2400088423; https://www.chictr.org.cn/showproj.html?proj=238565, accessed on 7 May 2026).

### 2.2. Participants and Research Personnel

#### 2.2.1. Participants and Sample Size

Recruitment posters titled “AI Psychotherapy Experience Recruitment” were disseminated online through multiple channels targeting young adults, primarily including university campus social media groups (e.g., QQ, WeChat), student organization forums, and the external social media platform Rednote. Applicants completed questionnaires and participated in pre-intervention interviews to assess their treatment motivation and determine eligibility. While this study is exploratory in nature and not designed as a confirmatory trial, sample size estimation was performed using G*Power 3.1 software (Heinrich-Heine-Universität Düsseldorf, Düsseldorf, Germany) for an ANOVA with repeated measures (within-between interaction): effect size (f) was set at 0.30, derived from a recent meta-analysis of AI-based randomized controlled interventions for anxiety [33], power (1 − β) at 0.80, and significance level (α) at 0.05, number of groups = 3, number of measurements = 3, and a correlation among repeated measures of 0.5. Based on these parameters, a minimum of 75 participants were required to achieve the desired statistical power. A final goal sample size was set at 84, accounting for 10% attrition rate and potential confounding factors.

Inclusion criteria: (1) Age 18 to 30 years old; (2) Proficient in using smartphones or tablets and communicating in Chinese; (3) Agreement to complete follow-up questionnaires and allow for the export of therapy chat records; (4) Generalized anxiety disorder 7-item scale (GAD-7) score above 5 and less than or equal to 21. Exclusion criteria: (1) Severe physical or organic brain diseases; (2) History of substance abuse (e.g., alcohol); (3) Major depressive episodes or suicidal attempts identified during the pre-intervention interview within one month prior to the trial; (4) Currently receiving other psychological or pharmacological treatment; (5) History of diagnosed mental illness.

The broader research project was pre-registered with the Chinese Clinical Trial Registry, which outlined the recruitment for a larger sample of young adults intended for multiple analyses. The present study constitutes a post hoc, exploratory analysis of an anxiety-focused subsample drawn from this larger pool. To ensure procedural transparency, we specify that the inclusion/exclusion criteria for this secondary screening were finalized in April 2024, after the initial data collection for the larger project was complete but prior to any formal data analysis or accessing of the outcome data for this specific subsample. This secondary screening applied the inclusion/exclusion criteria detailed herein to identify a suitable subsample for this anxiety-focused investigation. Consistent with the specific focus of this paper, findings related to other outcomes from the larger project (such as the PHQ-9) will be reported in a subsequent paper.

As part of the study’s safety protocols, all participants (Control group included) were provided with the phone number of the local free mental assistance hotline to facilitate immediate intervention in the event of a psychological crisis. Any adverse events, such as a significant worsening of psychological distress or suicidal ideation reported by participants or identified by the research team, were to be documented and immediately addressed. Throughout the trial, no such adverse events or harms were reported in any of the three groups.

#### 2.2.2. Research Team

Peer Counselors: 10 undergraduate students majoring in applied psychology and psychiatry served as peer counselors.

Research staff: The research staff consisted of 5 individuals, including 4 undergraduate students in applied psychology and 1 graduate student in applied psychology responsible for managing the trial. Staff coordinated all participant logistics and ensured that the single-blind procedures were maintained throughout the trial.

Supervisors and Clinicians: 1 professional supervisor with expertise in CBT provided guidance to the peer counselors. 2 licensed psychotherapists conducted pre- and post- interviews with the participants.

#### 2.2.3. Randomization and Blinding

Participants were stratified into three levels of anxiety severity based on their GAD-7 scores to ensure a balanced distribution of anxiety severity across the study groups: mild anxiety (5–9), moderate anxiety (10–14), and severe anxiety (15–21) [34]. Stratified randomization was performed using a computer-generated randomization list to randomly assign participants within each anxiety severity stratum to one of the three groups (AI group, Human group, or Control group). There was no private relationship established between the research staff and participants outside of the structured study interactions.

A single-blind design was implemented to minimize potential bias due to participant or interviewer expectations.

Participant blinding: Both intervention groups were informed that their therapist was AI to ensure consistent perceptions of the therapist’s identity. To maintain this illusion in the Human group, the following measures were taken: (1) Recruitment materials omitted any mention of peer counselors; (2) Participants were introduced to details about the study’s AI system. They were instructed that “any curiosity or questions unrelated to the therapy content could be addressed to the staff after the study, please focus on completing the therapy sessions”.

Interviewer blinding: The licensed psychotherapists conducting the pre-intervention interviews were blinded to participant group assignment throughout the study.

### 2.3. Intervention

Both interventions aimed to provide online support based on Cognitive Behavioral Therapy (CBT) principles. The core therapeutic principles, specific techniques, guiding principles for therapist delivery (differentiated for AI and Human modalities), and illustrative intervention examples applicable to both groups are detailed in the CBT Intervention Guidelines (see Supplementary Material S1). This document served as the common foundational framework for both intervention arms. However, it is important to note that unlike many manualized CBT studies, neither the AI nor the Human intervention in this study followed a predetermined, session-by-session structured content plan. Instead, while the therapists (both the AI chatbot and the peer counselors) were guided by/trained in CBT principles and techniques, the specific topics discussed and the flow of each session were largely responsive to the participant’s immediate concerns and input, allowing for considerable flexibility and participant direction.

#### 2.3.1. AI Group

A therapeutic chatbot was employed for the trial. Deployed on QQ (the most popular instant messaging platform in China), the chatbot was powered by Microsoft Copilot (formerly Bing Chat, Microsoft Corporation, Redmond, WA, USA), which utilized the GPT-3.5-Turbo model. The specific version accessed during the study was the one publicly available in October 2023, with the underlying model having a knowledge cutoff of September 2021. Participants in the AI group received intervention through this chatbot, accessible via the QQ mobile application available on Windows, macOS, Android, and iOS. To ensure standardized intervention timing, researchers were responsible for managing access to the AI chatbot, strictly limiting interactions to the scheduled session times. Participants could not interact with the AI chatbot outside these designated periods.

The chatbot was developed using ChatGPT-Plugin, an open-source project on GitHub (GitHub, Inc., San Francisco, CA, USA). Its role as a CBT-oriented therapist was defined using custom settings. Specifically, the AI’s configuration and response generation were guided by a detailed Prompt Engineering Framework (see Supplementary Material S2). This framework operationalized the core principles and techniques outlined in the shared CBT Intervention Guidelines (see Supplementary Material S1), drawing upon several professional CBT books [35,36] and expert evaluation to ensure adherence to CBT principles in its text generation. The intervention consisted primarily of multi-turn dialogues and personalized customization. During these dialogues, Participants interacted with the chatbot by typing text messages, which it processed using natural language understanding and deep learning [25] to generate appropriate responses. Similar to a human-delivered CBT session, the chatbot’s primary tasks throughout the intervention were to: (1) gather information about participants’ specific psychological distress; (2) guide them to identify and separate their emotions, thoughts, and behaviors; and (3) encourage the development of new, more adaptive thought patterns and behaviors through evidence-based CBT techniques including cognitive restructuring, behavioral activation, and relaxation techniques. For participants who demonstrated a good understanding of core CBT principles, as assessed through weekly quizzes and therapist feedback, the later stages of the intervention involved exploration of personal growth areas such as identifying values, setting meaningful goals, and developing strategies for improving interpersonal relationships. The chatbot facilitated these explorations through guided self-reflection exercises and evidence-based techniques such as values clarification and behavioral experiments. In addition, rules governing the chatbot’s initiation and termination of conversations were established by the research team. Participants initiated and ended each therapy session by sending messages to the chatbot. To personalize the intervention, basic demographic and clinical information (age, gender, occupation, anxiety levels, and descriptions of psychological distress) was collected during pre-intervention interviews and incorporated into the chatbot’s setting for each participant.

However, recognizing the potential limitations of AI and prioritizing participant well-being, several safeguards were implemented to ensure ethical and appropriate interaction. If the chatbot’s response was off-topic or unhelpful, built-in backup responses were used to redirect the conversation or offer general encouragement. Participants were provided with clear instructions on how to report any concerning chatbot responses to the research team through a dedicated communication channel. To further mitigate potential risks, the research team actively monitored all chatbot interactions. In any instance where the chatbot’s response indicated potential emotional distress, risk of harm to oneself or others, or any other ethically concerning content, the research team immediately intervened, providing direct support to the participant and, if necessary, referring them to appropriate mental health professionals.

#### 2.3.2. Human Group

The human intervention was delivered by peer counselors [37] (trained and supervised undergraduate students), an approach chosen for its feasibility [37], accessibility [38], and alignment with the preferences of young adults [39].

To ensure the quality of the peer counseling delivered in this study, thirty undergraduate students majoring in applied psychology and psychiatry were recruited as potential peer counselors. They underwent a rigorous three-month training and selection process, the details of which are outlined in the Peer Counselor Training and Supervision Manual (see Supplementary Material S3). This process included didactic instruction, simulated AI client interactions, peer supervision, role-playing with standardized patients, and ongoing clinical supervision.

(1) Didactic Instruction: Trainees received instruction on the fundamental principles, processes, and techniques of CBT. Case examples were used to illustrate the application of CBT principles in real-world scenarios.

(2) AI Client Interaction: Trainees engaged in four simulated counseling sessions with an AI chatbot acting as the client. Each session focused on a different case scenario (e.g., family relationships, academic stress, relationship issues). After each session, trainees documented their peer supervision analysis of the case, providing a written summary of their reflections and insights.

(3) Peer Supervision: Following each AI client interaction, trainees engaged in peer supervision, working in pairs to analyze and reflect on the session. This process included case presentations, identification of challenges, and collaborative problem-solving.

(4) Role-Playing with Standardized Patients: Trainees participated in role-playing exercises with standardized patients portraying clients with anxiety symptoms. This component spanned four weeks, with two sessions per week, simulating the format and duration of the actual study intervention. Following the role-playing, each trainee submitted a comprehensive case report.

(5) Standardized Competency Assessments: Trainees’ competency in delivering iCBT was assessed using standardized measures after the practical experience component.

Training also focused on familiarizing them with AI communication styles and enhancing their online text-based intervention skills. Ten students who met the competency criteria were selected to serve as peer counselors. During the intervention, ongoing clinical supervision from a licensed professional with over ten years of experience in psychotherapy ensured adherence to iCBT protocols.

### 2.4. Instruments

#### 2.4.1. Measures

Anxiety levels were assessed using the Generalized Anxiety Disorder 7-Item Scale (GAD-7) [34], a widely used self-report measure based on *the Diagnostic and Statistical Manual of Mental Disorders*, fourth edition criteria (DSM-IV). This 7-item scale uses a 4-point Likert scale (0–3) for each item, with total scores ranging from 0 to 21. The scores are interpreted as follows: 0–5 (minimal), 6–9 (mild), 10–14 (moderate), and 15–21 (severe). Cronbach’s alpha for the GAD-7 in this study was 0.850.

The Chinese version of the Self-Efficacy for Exercise Scale (SEE-S) was used to measure participants’ self-efficacy for exercise [40]. This 9-item scale uses a 10-point Likert scale (0 = not at all confident, 10 = very confident) to assess perceived capability to exercise in various challenging situations (e.g., bad weather, feeling depressed). Higher scores indicate greater self-efficacy for exercise. Cronbach’s alpha for the SEE-S in this study was 0.873.

The Pittsburgh Sleep Quality Index (PSQI), a self-report questionnaire [41], was used to assess sleep quality. It comprises 18 items, grouped into seven components: subjective sleep quality, sleep latency, sleep duration, habitual sleep efficiency, sleep disturbances, use of sleeping medication, and daytime dysfunction. Each item is scored on a 4-point Likert scale (0–3), with total scores ranging from 0 to 21. Higher scores indicate poorer sleep quality. Cronbach’s alpha for the PSQI in this study was 0.702.

The Psychotherapy Benefit Scale (PBS), based on the Outcome Questionnaire-45 (OQ-45) [42] and the Chinese Health Questionnaire-12 (CHQ-12) [43], was designed to assess participants’ subjective perceptions of therapy benefits and outcomes. It encompasses three dimensions: well-being, functional improvement, and hope and expectations. This 15-item scale is rated on a 5-point Likert scale (1 = strongly disagree, 5 = strongly agree). Higher scores reflect more positive treatment experiences and perceived benefits. The scale demonstrated good internal consistency in this study, with a Cronbach’s alpha of 0.962 for the total scale and alphas ranging from 0.826 to 0.937 for the three subscales.

#### 2.4.2. Psychotherapy System Information Sheets

Two information sheets, titled “Introduction to the AI Psychotherapy System” and “Introduction to the Online Psychotherapy System,” were developed for the AI and Human groups, respectively. These sheets aimed to familiarize participants with the intervention systems, standardize the intervention procedures, and enhance trust in the therapist. Both information sheets omitted identifying information about the intervention providers and presented identical intervention procedures to minimize potential bias due to the inherent differences between therapists.

#### 2.4.3. Pre-Intervention Interview

*The Mini-International Neuropsychiatric interview* (MINI) was used as a pre-intervention interview [44]. The MINI is a brief structured clinical interview designed to screen and diagnose 16 axis I psychiatric disorders according to DSM-IV and ICD-10 criteria. In this study, licensed psychotherapists assessed participants’ psychological status using the three modules of the MINI for generalized anxiety disorder, major depressive episode, and suicidality.

#### 2.4.4. Post-Intervention Interview

The Interpersonal Process Recall (IPR) method was used for post-intervention interviews [45]. These interviews followed a semi-structured format guided by a specific interview guide, which can be found in Supplementary Material S4. IPR is a collaborative interview approach often employed in psychotherapy research. After completing the intervention, participants reviewed their intervention session chats with the researcher to facilitate reflection on their thoughts, feelings, and experiences during the intervention.

### 2.5. Study Procedure

#### 2.5.1. Pilot Study

A pilot study involving 6 participants (3 in the AI group and 3 in the Human group) was conducted. Participants were informed that they were participating in “a study about AI psychotherapy.” They engaged in text-based intervention sessions and post-intervention interviews, which included a manipulation check to assess the effectiveness of the experimental manipulation. During the interview, participants were asked verbally: “During your interactions with the AI counselor, to what extent did you feel you were communicating with a machine versus a human? Please elaborate on your reasoning.” The full interview guide including this question and a follow-up on perceived comprehension accuracy is available in Supplementary Material S3, Part 2, Question 3. Results from the manipulation check indicated that participants in the Human group did believe their therapist was AI. Additionally, the pilot study’s post-intervention interviews were used to refine the interview outline for the main study.

#### 2.5.2. Formal Study

Interested individuals completed screening questionnaires. Eligible participants provided informed consent and underwent pre-intervention interviews with licensed psychotherapists to assess their psychological status. The intervention phase lasted for four weeks. Participants in the AI group received AI-CBT from the chatbot, while those in the Human group received Human-CBT from peer counselors. Both groups received two one-hour intervention sessions per week for a total of eight hours of intervention.

All participants completed the SEE-S and PSQI scales weekly following their intervention sessions. In the second and fourth week, participants completed the GAD-7 again. The GAD-7 was re-administered in the second and fourth weeks to track changes in anxiety levels. After the four-week intervention, participants completed the PBS to assess their perceived benefits from the intervention. Finally, the research staff conducted individual post-intervention interviews with each participant, audio-recording the sessions to gather qualitative data on their experiences. These interviews also included a manipulation check to assess the effectiveness of the participant blinding. Crucially, in the Human group, none of the participants spontaneously expressed suspicion that their therapist might be a human prior to the debriefing, suggesting the blinding procedure was successfully maintained throughout the main trial.

#### 2.5.3. Debriefing

After completing the study, all participants attended a debriefing session during which the rationale for the study design, including the use of peer counselors posing as AI in the human intervention group, was thoroughly explained. Participants were offered the opportunity to ask questions and discuss any concerns or feelings they had about the study design.

### 2.6. Data Analysis

Baseline demographic and clinical characteristics were compared across the three groups (AI, Human, and Control) using one-way analysis of variance (ANOVA) for continuous variables and chi-square tests for categorical variables. The primary outcome, anxiety levels as measured by the GAD-7, was analyzed using a two-way repeated measures ANOVA based on the intention-to-treat (ITT) principle (*n* = 90). Missing data for the three participants who dropped out were handled using the Last Observation Carried Forward (LOCF) method. A sensitivity analysis using the per-protocol sample (*n* = 87) was also conducted. Secondary outcomes, including self-efficacy for exercise (SEE-S) and sleep quality (PSQI), were analyzed using Generalized Estimating Equations (GEEs). As the three participants who dropped out had no data for these secondary outcomes, these analyses were conducted on the available complete cases (*n* = 87). A different analytical framework was chosen for these secondary outcomes to better accommodate their distinct measurement structure. Specifically, for the primary outcome with three fixed time points, the conventional repeated-measures ANOVA provided a powerful and straightforward analysis. However, for the secondary outcomes measured more frequently (four time points), GEEs offered a more robust and flexible approach to model the within-subject correlation structure over time. Independent samples *t*-tests were conducted to compare post-intervention scores on the Psychotherapy Benefit Scale (PBS) between the AI and Human intervention groups.

Qualitative data were analyzed using NVivo 12 Plus (QSR International, Burlington, MA, USA) following a thematic analysis approach. To ensure coding reliability, all transcripts were independently coded by two researchers. Inter-rater reliability was substantial, with Cohen’s Kappa coefficient of 0.83 calculated on 25% of the data. All coding discrepancies were resolved through discussion with a senior researcher to reach consensus. The finalized codes were then grouped and conceptually integrated to develop the overarching themes.

## 3. Results

### 3.1. Participant Flow

Of 388 individuals who responded to the recruitment poster, 90 met the eligibility criteria and were enrolled in the study. In total, 87 participants completed the study, resulting in an attrition rate of 3.3% (3/90). Most participants were students (*n* = 82), with smaller proportions employed (*n* = 5) or categorized as ‘other’ (*n* = 3). Participants’ ages ranged from 18 to 27 years (M = 20.41, SD = 1.92).

### 3.2. Baseline Data

One-way ANOVA was conducted to compare baseline characteristics between the three groups. As shown in Table 1, no significant differences were found in age, gender ratio, or anxiety levels, indicating that the randomization procedure was successful in creating comparable groups.

### 3.3. Quantitative Analysis

#### 3.3.1. Changes in Anxiety Levels: Within-Group and Between-Group Comparisons

A two-way repeated measures ANOVA was conducted to examine changes in anxiety levels over time and among groups. The data met the assumptions of normality (Shapiro–Wilk test) and sphericity (Mauchly’s W = 0.990, *p* = 0.638).

The primary analysis was conducted on the ITT sample (*n* = 90). The repeated measures ANOVA revealed a significant interaction effect between group and time F = 3.569, *p* = 0.008, η^2^ = 0.076). The main effect of group was not significant (F = 1.385, *p* = 0.256, η^2^ = 0.031), while the main effect of time was significant (F = 18.797, *p* < 0.001, η^2^ = 0.178).

Simple effects of group were examined at each time point. At baseline (T0) and week 2 (T2), there were no significant differences in anxiety levels among the three groups. At post-intervention (T4), the effect of group on anxiety levels did not reach statistical significance in the ITT analysis (F = 2.746, *p* = 0.070, η^2^ = 0.59). Post hoc pairwise comparisons confirmed that neither the Human group (*p* = 0.091) nor the AI group (*p* = 0.227) showed a statistically significant reduction in anxiety compared to the Control group at T4.

Sensitivity Analysis. To assess the robustness of our findings to attrition, a secondary per-protocol (PP) analysis was conducted on the 87 participants who completed the entire study. In the PP analysis, the interaction effect remained significant (F = 4.152, *p* = 0.003, η^2^ = 0.090). Notably, the effect of group at T4 was significant in the PP sample (F = 4.780, *p* = 0.011, η^2^ = 0.102), with pairwise comparisons revealing that the Human group had significantly lower anxiety levels compared to the Control group (*p* = 0.009).

Simple effects of time: time had a significant effect on anxiety levels in both the AI group (F = 6.845, *p* = 0.002, η^2^ = 0.142) and the Human group (F = 17.394, *p* < 0.001, η^2^ = 0.295), but not in the Control group (F = 1.778, *p* = 0.175, η^2^ = 0.041). Pairwise comparisons (see Table 2) revealed that the AI group’s anxiety levels decreased significantly from baseline (T0) to the midpoint (T2, *p* = 0.003), but did not decrease further from the midpoint to post-intervention (T4), suggesting a plateau effect. In contrast, the human group showed a consistent pattern of anxiety reduction throughout the study, with anxiety levels at T0, T2, and T4 all being significantly different from each other (all *p*s < 0.05). As expected, the control group did not show significant changes in anxiety levels over time.

#### 3.3.2. Changes in Self-Efficacy for Exercise and Sleep Quality

GEEs were used to examine changes in self-efficacy for exercise and sleep quality over time. Details of the parameter estimates for both outcomes are presented in Table 3.

For self-efficacy for exercise, there was no significant interaction between group and time (Wald = 7.670, *p* = 0.263), suggesting that the two interventions had comparable effects on self-efficacy for exercise across the four weeks. While the effect of group was not significant, time did have a significant positive effect (β > 0). Participants in both intervention groups showed increases in self-efficacy for exercise over time, with significant improvements observed from week 1 to both week 3 and week 4 (*p* < 0.05).

The analysis of sleep quality revealed a similar pattern. There was no significant group-by-time interaction (Wald = 7.751, *p* = 0.257), indicating that the effects of the interventions on sleep quality did not differ significantly across the four weeks. As with self-efficacy for exercise, the effect of group was not significant, but the effect of time was (β < 0). Participants in both intervention groups showed improvements in sleep quality over time, with significant differences between week 1 and weeks 2, 3, and 4 (*p* < 0.05).

In summary, while both interventions led to improvements in self-efficacy for exercise and sleep quality, these changes were not significantly different between the AI and Human groups.

#### 3.3.3. Differences in Perceived Psychotherapy Benefit

An independent samples *t*-test was conducted to compare scores on the Psychotherapy Benefit Scale between the AI and Human groups. The Human group (Mean = 63.67, SD = 7.41) reported significantly higher perceived benefit from the intervention compared to the AI group (Mean = 53.97, SD = 12.31; t = −3.55, *p* < 0.001, 95% CI: −15.17 to −4.23).

#### 3.3.4. Qualitative Analysis

Semi-structured interviews were conducted using the IPR method, guided by grounded theory principles [46]. The interview outline included three main sections: (1) “Please describe your thoughts and feelings during the intervention process.”; (2) “If you had been informed beforehand that your therapist was not AI but human, how do you think it would have been different?”; (3) “What are your thoughts on AI being used in psychotherapy and entering into people’s intimate relationships?”. Three major themes emerged from the qualitative analysis, representing key aspects of participants’ experiences with AI and human interventions: intervention process, intervention outcomes, and future perspectives. Table 4 provides a detailed overview of the subthemes identified within each major theme.

#### 3.3.5. Intervention Process

The two intervention groups exhibited distinct strengths. Participants who received the AI intervention highlighted its convenience, noting the ease of overcoming time and location constraints (A1), reduced privacy concerns (A2), and diminished feelings of stigma associated with seeking mental health support (A3). Consequently, they felt more comfortable disclosing personal information and appreciated the time-saving benefits of the AI intervention. Additionally, the AI intervention provided access to a vast pool of information (A5) and demonstrated flexibility in navigating different conversation topics (A6). In contrast, the human intervention excelled in fostering a sense of interpersonal connection and demonstrating superior semantic comprehension, resulting in richer emotional exchanges (A6).

The AI intervention struggled with recognizing the nuances of emotional language, hindering its ability to fully grasp emotional complexities and provide adequate emotional support (A7). AI-generated suggestions were sometimes perceived as idealistic and impractical (A8). Participants also raised concerns about the formulaic nature of the AI’s intervention approach (A11), its mechanistic response style (A10), and instances of unproductive probing (A12). While both intervention groups exhibited limitations in their capacity for empathy, these limitations manifested differently. The AI group’s empathy was constrained by the technology’s limited ability to perceive and respond to emotions, resulting in fewer empathic responses (A9). The human therapists’ ability to convey empathy, while present, was somewhat restricted by the text-based communication medium (A7) and slow response time (A11), limiting the depth of emotional expression they could convey. Interestingly, the Human group’s response style also displayed some degree of mechanization, likely due to the peer counselors’ efforts to simulate AI communication patterns (A10).

#### 3.3.6. Intervention Effectiveness

Both AI and human interventions were effective in providing practical advice tailored to participants’ presenting concerns (A13), and both groups reported similar levels of satisfaction with the overall effectiveness of the interventions (A16). However, human intervention was perceived as superior in providing emotional support (A14) and facilitating deeper self-reflection (A15). The human therapists were viewed as more adept at understanding participants’ perspectives, validating their emotions, identifying irrational beliefs, and introducing new possibilities.

#### 3.3.7. Future Perspectives

Participants generally expressed a preference for AI interventions due to their perceived personalization, accessibility, and stability (A18). However, human interventions were still regarded as valuable in certain contexts, particularly for their perceived expertise and ability to provide richer empathy and content (A19). Since both groups believed they were interacting with AI, they expressed optimism about the future applications of AI-driven interventions (A20), particularly for addressing mild to moderate mental health concerns and providing short-term psychological support (A21).

## 4. Discussion

### 4.1. Current Findings

This study’s findings present a nuanced and cautious picture of the effectiveness of both AI- and peer-counselor-delivered CBT. Our primary ITT analysis revealed that while participants in both intervention groups exhibited significant pre- to post-intervention reductions in anxiety, neither intervention was statistically superior to the no-intervention control group. This conservative but robust finding stands as the central result of our trial, tempering optimistic claims about the immediate clinical efficacy of these brief, online interventions. Interestingly, a secondary PP analysis on study completers did reveal a significant benefit for the peer-counselor group (*p* = 0.009). The divergence between the ITT and PP results can likely be attributed to the statistical impact of the small number of dropouts (*n* = 3) in this moderately sized trial, suggesting that the benefits of the peer-counselor intervention may be most pronounced for individuals who complete the full course of therapy. This complex pattern—between promising within-group effects and a lack of definitive between-group efficacy—forms the central tension that this discussion aims to explore.

Beyond statistical significance, the clinical relevance of these changes also warrants consideration. The mean reduction in GAD-7 scores for the peer-counselor group (3.57 points in ITT, 3.96 in PP) approached or met the threshold for a minimally clinically important difference (MCID) of approximately 3–4 points [47]. In contrast, the change in the AI group (1.94 points) likely fell below this threshold. This suggests that while both interventions showed some benefit, only the peer-counselor intervention demonstrated the potential to produce a change that users might perceive as clinically meaningful.

The observed plateau effect in the AI group, coupled with its lack of superiority over the control group, can be understood through several factors. Firstly, although the AI was designed via prompt engineering to adhere to CBT principles, our findings suggest that the AI was perceived by participants as having limitations in accurately recognizing and responding to complex emotions or interpreting nuanced contextual cues. This aligns with our qualitative findings, where some participants in the AI group reported experiences of the AI lacking deeper understanding or emotional resonance. Secondly, the AI’s interaction style was frequently described by participants as relatively formulaic in certain situations, particularly during deeper explorations touching upon core beliefs within the unstructured dialogue format, which might necessitate more flexible and personalized responses. As mentioned by some participants in interviews, this interaction style sometimes might not have effectively facilitated deep self-reflection or could even evoke negative emotional experiences. Furthermore, this perceived lack of understanding or the limitations in interaction style might have contributed to decreased motivation for some participants in the later stages of the intervention, potentially explaining the significantly lower scores on the Psychotherapy Benefit Scale (PBS) in the AI group, and ultimately leading to the leveling-off of anxiety reduction. Therefore, these findings suggest that enhancing the effectiveness of large language models guided solely by prompt engineering in psychotherapy, particularly in generating responses that are perceived as more emotionally attuned in unstructured dialogues, may require further exploration and optimization in areas such as prompt strategy refinement, adaptability to individual needs, and the ability to simulate more naturalistic interactions, rather than solely relying on general advancements in AI technology.

An intriguing finding was that while both intervention groups showed improvements in sleep quality and exercise self-efficacy over time, these changes were not superior to the control group. As noted by the reviewer, this may suggest an influence of non-specific factors, such as measurement effects or participation itself (i.e., the Hawthorne effect) [48]. A complementary interpretation, however, is that these health behaviors may be an indirect consequence of the primary reduction in anxiety. A large body of literature demonstrates a strong, bidirectional link between sleep disturbances and the cognitive components of anxiety, such as worry, rumination, and obsessive-compulsive symptoms [49,50]. It is plausible that even the modest anxiety reduction observed in our intervention groups could have downstream benefits on sleep by reducing pre-sleep cognitive arousal. The lack of a significant between-group effect could then be explained by the modest overall efficacy of the interventions. This finding is particularly relevant for university student populations, who often experience a complex interplay between academic stress, sleep patterns, and mental health [51].

Our qualitative analysis of participants’ perspectives on the future of AI interventions also revealed distinct preferences and expectations. Those who favored AI interventions appreciated their cost-effectiveness, availability, and the sense of privacy it afforded. Conversely, those who preferred the peer-counselor intervention emphasized the importance of professional expertise, the capacity for deep emotional connection, and the richness of human interaction and insight. While some participants expressed a desire for wider access to AI interventions, they also voiced reservations about the maturity and trustworthiness of current AI models. Despite these concerns, participants generally agreed that AI interventions, in their current form, appear best suited for addressing mild-to-moderate life or work-related challenges and providing readily available, short-term psychological support. This suggests a potential role for AI as a scalable, first-line support tool within a stepped-care model, where users could be escalated to human-led care if needed.

### 4.2. Limitations

Several limitations of our study warrant consideration. First and foremost, the study’s primary limitation is its exploratory nature, stemming from the post hoc selection of the analytic subsample. As detailed in the introduction, this discrepancy from the pre-registration introduces a high and unquantifiable risk of selection bias that cannot be fully ruled out. For instance, this procedure may have inadvertently selected for participants who were more motivated or amenable to therapy, potentially inflating the observed treatment effects. Consequently, the findings, particularly the efficacy results, must be interpreted with significant caution and should be viewed as preliminary and hypothesis-generating. Crucially, the necessary next step is to replicate these findings in a methodologically robust, prospectively registered confirmatory trial.

Second, our operationalization of the interventions introduces specific limitations. Our reliance on a single AI chatbot (ChatGPT-3.5, OpenAI, San Francisco, CA, USA) may limit the generalizability of our findings to other AI models with diverse conversational styles. Additionally, the non-manualized, flexible design of both interventions, while intended to enhance participant engagement, introduces variability and limits strict replicability. Furthermore, the deception-based design for the human group, while intended to control for expectancy effects, likely constrained the effectiveness of the peer-counselor intervention. By instructing peer counselors to simulate an AI’s communication style, we may have inadvertently suppressed the very qualities—such as spontaneous empathy and flexible rapport-building—that define high-quality human-led therapy. The qualitative data, where some participants in the peer-counselor group noted a ‘mechanistic’ style, supports this interpretation. Therefore, the observed efficacy difference between the two intervention groups might represent an underestimation of the true potential gap between genuine human therapy and current AI capabilities.

Third, while our qualitative findings offer valuable context, the lack of quantitative process measures (e.g., alliance ratings, cognitive measures) limits our ability to rigorously test the specific mediational pathways (e.g., therapeutic alliance, cognitive change) that may explain the observed group differences in outcomes and perceived benefit.

Finally, the generalizability of our findings may be limited by the characteristics of our sample. Our reliance on a university-based sample of young adults with mild-to-moderate anxiety (based on the GAD-7 cutoff score) means the results may not extend to clinical populations with more severe or comorbid conditions, or to different age groups. Future research should replicate this trial with more clinically diverse samples [52,53].

## 5. Conclusions

In conclusion, this exploratory randomized controlled trial, analyzed using a conservative intention-to-treat approach, demonstrates that while both AI- and peer-counselor-delivered CBT show within-group promise for anxiety reduction, the current evidence does not support their superiority over a no-intervention control in young adults. Our mixed-methods analysis suggests the efficacy gap between the two active interventions is driven by the AI’s limitations in providing the flexible, emotionally supportive, and personalized interaction that participants value. These findings highlight that while scalable digital interventions can engage users and lead to some improvement, claims of their clinical efficacy must be made with significant caution. Future prospectively registered trials are essential to validate these preliminary findings and to determine the conditions under which AI can be most effective.

## Figures and Tables

**Table 1:** Baseline Characteristics of the participants (*n* = 90).

Variables	AI Group	Human Group	Control Group	*F*/χ^2^	*p*
Age, mean (SD), years	20.37 (1.83)	20.23 (2.32)	20.60 (1.63)	0.271	0.763
Gender, *n* (%)					
-Male	8 (26.67)	12 (40.00)	6 (20.00)	3.029	0.220
-Female	22 (73.33)	18 (60.00)	24 (80.00)		
Employment, *n* (%)					
-Students	27	27	28	4.824	0.306
-Employed	3	2	0		
-Others	0	1	2		
Scale, mean (SD)					
-GAD-7	7.97 (2.77)	9.17 (3.54)	8.77 (3.70)	0.990	0.376
Anxiety level, *n* (%)					
-Mild	21 (70.00)	17 (62.96)	20 (66.67)	2.494	0.646
-Moderate	7 (23.33)	11 (33.33)	9 (30.00)		
-Severe	2 (6.67)	2 (3.70)	1 (3.33)		

**Table 2:** Pairwise comparisons of anxiety levels over time (*n* = 90).

Group	Time Contrast	MD	SD	*p*
AI group	T0–T2	2.00	0.58	0.003
	T0–T4	1.93	0.64	0.010
	T2–T4	−0.07	0.61	1.000
Human group	T0–T2	1.40	0.58	0.056
	T0–T4	3.57	0.64	<0.001
	T2–T4	2.17	0.61	0.002
Control group	T0–T2	1.10	0.58	0.189
	T0–T4	0.80	0.64	0.639
	T2–T4	−0.30	0.61	1.000

Abbreviations: MD, mean difference; SD, standard deviation. Note: T0 = baseline; T2 = week 2; T4 = week 4.

**Table 3:** Parameter estimates from Generalized Estimating Equations (GEEs) for changes in exercise self-efficacy and sleep quality from week 1 to week 4 (*n* = 87).

Variables	Parameter	β ^a^	SE	*p*	95% CI	
					Lower	Upper
Self-efficacy for exercise	Intercept	34.71	4.30	<0.001	26.28	43.14
	T4	7.92	1.53	<0.001	1.53	4.93
	T3	6.08	1.20	<0.001	3.73	8.44
	T2	0.10	1.01	0.918	−1.87	2.08
	T1	0 ^b^				
	AI group	−6.12	7.37	0.406	−20.56	8.33
	Human group	−0.78	6.71	0.908	−13.94	12.38
	Control group	0 ^b^				
Sleep quality	Intercept	7.44	0.54	<0.001	6.37	8.50
	T4	−0.95	0.37	0.010	−1.68	−0.23
	T3	−0.91	0.34	0.008	−1.58	−0.23
	T2	−1.25	0.29	<0.001	−1.83	−0.68
	T1	0 ^b^				
	AI group	0.54	0.71	0.448	−0.86	1.94
	Human group	−0.01	0.74	0.993	−1.46	1.45
	Control group	0 ^b^				

Abbreviations: SE, standard error; CI, confidence interval. Note: T1 = week 1; T2 = week 2; T3 = week 3; T4 = week 4. ^a^ regression coefficient. ^b^ This parameter is set to zero as the reference category.

**Table 4:** Qualitative themes and subthemes: AI intervention vs. human intervention.

Theme	Subtheme	AI Group ^c^	Human Group	Concept
Process (Advantages)	A1: Reduced time/location constraints	8	7	Cost-effective, saves time
	A2: Reduced privacy concerns	15	9	Safe environment, increased self-disclosure
	A3: Reduced stigma	14	10	Reduced psychological burden
	A4: Sense of interaction	11	16	Provides an outlet for sharing or feeling heard
	A5: Broad information resources	11	0	Extensive information coverage
	A6: Comprehension ability	15	14	Flexible counseling direction (10/5) ^d^ captures semantics and key issues (5/9)
(Disadvantages)	A7: Weak empathy	17	16	Limited ability to understand circumstances (3/11); identify emotional fluctuations, and provide emotional validation (14/5)
	A8: Idealistic advice	11	8	Impractical suggestions
	A9: Weak recognition ability	5	0	Limited capacity to process language and perceive emotions
	A10: Mechanistic language	15	21	Inflexible and impersonal tone and response style
	A11: Weak sense of communication	34	53	Formulaic counseling style (34/0); slow response time (0/53)
	A12: Unproductive probing	3	1	Lack of direction in guidance
Effectiveness	A13: Providing advice	25	20	Offers suggestions for practical problems
	A14: Emotional support	8	18	Understands client’s perspective and improves mood
	A15: Thought-provoking	18	30	Identifies irrational beliefs and introduces new possibilities
	A16: Achieved desired outcomes	21	20	Met expectations for counseling outcomes
	A17: Partially helpful	9	8	Only addressed surface-level issues
Future	A18: Preference for AI	18	15	Personalized matching, accessibility, stability
	A19: Preference for human	9	6	Professionalism, emotional needs met, rich content exploration
	A20: Application and promotion	4	9	widespread adoption and dissemination
	A21: Scope of application	18	30	Suitable for mild to moderate problems, short-term interventions

Note: ^c^ The number represents the frequency of consistent content related to the corresponding subtheme obtained from node materials of different texts. Some nodes may contain multiple subthemes, and participants may have emphasized certain subthemes repeatedly, resulting in multiple counts for a single subtheme. ^d^ Numbers in parentheses represent the frequency with which AI and human interventions were specifically mentioned for distinct dimensions identified within certain concepts. The format is (AI group count/Human group count).

## Data Availability

The data presented in this study are not publicly available due to privacy and ethical restrictions. The dataset contains potentially identifying information on human participants, and sharing it publicly would compromise the confidentiality assured to them in the informed consent process. The data that support the findings of this study are available from the corresponding author, Xueling Yang, upon reasonable request, subject to a data sharing agreement and approval from the institutional review board.

## Supplementary Materials

The following supporting information can be downloaded at: https://www.mdpi.com/article/10.3390/healthcare14101325/s1, Supplementary Material S1: CBT Intervention Guidelines; Supplementary Material S2: Prompt Engineering Framework for AI Chatbot; Supplementary Material S3: Peer Counselor Training and Supervision Manual; Supplementary Material S4: Semi-structured Interview Guide.
