# Supplementary material for: AI Versus Human-Delivered Online Cognitive Behavioral Therapy for Anxiety Symptoms in Young Adults: A Randomized Controlled Trial
Source: Healthcare (Basel). 2026 May 13;14(10):1325. doi: 10.3390/healthcare14101325 (PMC13206094; doi:10.3390/healthcare14101325)
Supplement: Supplementary file 1 [file healthcare-14-01325-s001.zip › Supplementary Material 1:CBT intervention guidelines-tracked.pdf]

Supplementary Material 1: CBT Intervention Guidelines

Intervention Format: Text-based Internet Cognitive Behavioral Therapy (iCBT).

Intervention Duration: 4 weeks, with 2 interactions per week, each lasting approximately 60 minutes.

Part 1: Core Principles

1. Psychoeducation:

   Detailed Explanation: Systematically introduce participants to the core concepts and principles of CBT, including the cognitive model (the dynamic interaction between thoughts, emotions, and behaviors), the cognitive and behavioral maintenance mechanisms of psychological problems, and the crucial role of cognitive restructuring and behavioral change in improving mental health. Emphasize that CBT is a proactive, problem-focused, and time-limited therapeutic approach aimed at empowering participants with self-help skills.

   Goal: Provide participants with a clear understanding of CBT, establish expectations for the therapeutic process, and enhance their motivation and confidence in participating in therapy.

2. Cognitive Identification:

   Detailed Explanation: Guide participants in identifying and monitoring their automatic thoughts (unexamined, rapid thoughts in specific situations), core beliefs (deep-seated assumptions about themselves, others, and the world), and common cognitive biases (systematic errors in thinking, such as overgeneralization, catastrophizing, selective abstraction, all-or-nothing thinking, mind-reading, negative prediction, discounting the positive, emotional reasoning, "should" statements, and personalization). Emphasize the importance of recording thoughts for subsequent evaluation and challenge.

   Goal: Enhance participants' awareness of their own thinking patterns and identify specific cognitions associated with negative emotions and problematic behaviors.

3. Cognitive Restructuring:

Detailed Explanation: Teach participants to evaluate and question the identified negative or inaccurate thinking patterns. Guide them in examining the evidence supporting and contradicting these thoughts, and in identifying logical errors and biases in their thinking. Encourage participants to use techniques such as logical reasoning, reality testing, and positive reframing to develop more positive, balanced, and realistic alternative thoughts. Emphasize the importance of distinguishing between thoughts and facts.

Goal: Modify participants' dysfunctional thinking patterns, reduce the frequency and intensity of negative thoughts, and cultivate more flexible and adaptive ways of thinking.

4. Behavioral Activation:

Detailed Explanation: Encourage participants to actively engage in various activities that can bring positive emotions, a sense of accomplishment, and meaning. Emphasize the importance of activity scheduling and monitoring, even when feeling low, gradually increasing participation. Guide participants in identifying and overcoming barriers to activity engagement. Frame behavioral change as a crucial pathway to improving mood and thinking.

Goal: Break the vicious cycle between negative emotions and behavioral inactivity, increase positive experiences, elevate mood, and enhance self-efficacy.

5. Problem-Solving:

Detailed Explanation: Teach participants a systematic set of problem-solving steps, including identifying and defining the problem, brainstorming potential solutions, evaluating the pros and cons of each solution, selecting and implementing the best solution, and evaluating the outcome of the implemented solution. Emphasize breaking down complex problems into manageable smaller steps and fostering a proactive attitude towards challenges.

Goal: Enhance participants' ability to cope with life difficulties and challenges, reducing negative emotions arising from the inability to solve problems.

6. Emotion Regulation:

Detailed Explanation: Help participants identify and label their emotions,

understand the sources, functions, and expressions of emotions. Teach participants a range of healthy emotion management strategies, including cognitive reappraisal (changing how one thinks about an emotionally charged event), emotion acceptance, using relaxation techniques, engaging in positive activities, and developing more effective coping skills. Emphasize the importance of distinguishing between emotions and behavioral reactions.

Goal: Increase participants' awareness and understanding of their own emotions, teach them to manage emotions in healthier and more adaptive ways, and reduce emotional dysregulation and maladaptive behaviors.

7. Relapse Prevention:

Detailed Explanation: Help participants identify their potential triggers and early warning signs of relapse (e.g., the return of negative thoughts, decreased activity, sleep disturbances), and review the coping skills learned during therapy. Develop personalized relapse prevention plans, including identifying high-risk situations, warning signs, and coping strategies. Encourage participants to build a social support system and understand how to seek additional help if needed.

Goal: Help participants maintain therapeutic gains, enhance their ability to cope with future challenges, and reduce the risk of psychological problem recurrence.

Part 2: Guiding Principles for AI Group

1. Role Definition and Consistency:

Detailed Explanation: The "Xiao Zhi" chatbot is explicitly defined as a professional and trustworthy CBT therapist, with all interactions strictly adhering to the pre-set CBT principles and guidelines. Ensure consistency in the AI's role and responses throughout the intervention, avoiding behaviors or suggestions inconsistent with a therapist's identity.

Specific Operation: Through meticulous prompt engineering, ensure that the AI embodies the core concepts and techniques of CBT in all dialogues.

2. Empathy and Normalization:

Detailed Explanation: Despite being AI, express understanding and acceptance

of participants' emotions (empathy) through carefully designed language, and help participants recognize that their experiences and feelings are common to reduce feelings of loneliness and shame (normalization).

Specific Operation: Use pre-set empathic phrases and normalizing statements, such as: "I understand how you feel," "Many people experience similar feelings in these situations."

3. Structure and Step-by-Step Guidance:

Detailed Explanation: Strictly follow a pre-set, structured prompt engineering framework to guide participants through each stage of CBT and the learning of techniques. Ensure each interaction has clear goals and steps, progressing gradually.

Specific Operation: Use clear questions and instructions to guide participants through cognitive identification, evaluation, and challenge, as well as behavioral experiments and problem-solving tasks.

4. Balance between Automation and Personalization:

Detailed Explanation: Generate responses automatically based on natural language understanding and pre-set CBT guidelines, but where possible, incorporate a degree of personalization based on participants' previously provided information and current expressions to enhance relevance and engagement.

Specific Operation: Record and review participants' previous answers and refer to relevant information in subsequent interactions.

5. Standardization, Evidence-Based, and Traceability:

Detailed Explanation: Ensure that all CBT content and guidance received by participants are based on established CBT principles and clinical evidence. Each AI response should be traceable to the pre-set guidelines and theoretical foundations.

Specific Operation: Establish a clear prompt and knowledge base to ensure the AI's output aligns with CBT standard practices.

6. Ethics and Safety Assurance:

Detailed Explanation: Clearly inform participants about data privacy, security, and confidentiality measures at the beginning of the dialogue. When the AI detects expressions of self-harm or harm to others, it must immediately trigger the pre-set crisis

intervention protocol, providing emergency contact information and resources.

Specific Operation: Pre-set keyword trigger mechanisms and standardized crisis intervention responses.

Part 3: Guiding Principles for Human Group

1. Peer Support and Empowerment:

Detailed Explanation: Peer counselors, who have undergone professional CBT training, leverage their similar experiences and understanding to provide participants with emotional support, encouragement, and practical guidance. Emphasize empowering participants to develop self-help skills.

Specific Operation: Actively share personal experiences (within ethical and confidentiality boundaries), express understanding and encouragement.

2. Active Listening and Empathic Understanding:

Detailed Explanation: Listen attentively to participants' expressions, accurately understand their emotions and thoughts, and convey empathy and acceptance through words and tone. Establish a strong therapeutic alliance.

Specific Operation: Use open-ended questions, reflection, summarization, and emotional responses.

3. Personalization and Flexible Adaptation:

Detailed Explanation: Adapt the intervention content, pace, and focus flexibly based on participants' unique circumstances, needs, goals, and progress, while adhering to core CBT principles. Different techniques and methods can be chosen based on the specific situation.

Specific Operation: Pay attention to participants' feedback in each interaction and adjust the direction and depth of discussion according to their needs.

4. Encouraging Participation and Providing Positive Feedback:

Detailed Explanation: Actively encourage participants to engage in the therapeutic process, such as completing homework, sharing thoughts and feelings, and trying new behaviors. Provide timely positive feedback, acknowledging their efforts and progress.

Specific Operation: Use encouraging language, such as: "That's a great effort," "You're doing well," "That's an important step forward."

5. Maintaining Boundaries and Professional Responsibility:

Detailed Explanation: Counselors need to adhere to professional ethical guidelines, maintain clear counseling relationship boundaries, respect participants' privacy and autonomy. When identifywoyouing issues beyond the scope of peer support or high-risk situations, follow the pre-set referral procedures.

Specific Operation: Clearly define the scope and limitations of counseling, understand referral resources and procedures.

Part 4: Intervention Examples (Applicable to Both AI and Human Groups):

1. CBT Introduction and Cognitive Identification

Guidance: Hello! Welcome to this CBT intervention. CBT is an evidence-based psychotherapy approach that can help us understand the connection between thoughts, emotions, and behaviors, and learn practical skills to cope with psychological distress. Over the next four weeks, we will have two interactions per week, each lasting about an hour, where we will explore your thinking patterns, learn to manage emotions, and take positive steps to improve your life. Today, we will start by understanding your thoughts. Have you encountered any specific situations recently that have troubled you? Can you share what thoughts went through your mind at that time?”

Goal: Guide participants to describe specific distressing situations and identify at least one automatic thought that occurred in that situation. Introduce the basic concepts of the "thought-emotion-behavior" model.

2. Deeper Cognitive Identification and Emotion Connection

Guidance: In our last interaction, you mentioned [participant's previously shared thought]. Today, we will continue practicing identifying automatic thoughts in different situations and further explore the connection between these thoughts and the emotions you experienced. When you have the thought ‘[participant's previously mentioned thought]’, what emotions do you usually feel? Please describe them in a word or a few words. How intense are these emotions (for example, on a scale of 1 to 10, with 10

being the most intense)? What kind of relationship do you see between ‘[that thought]’ and your ‘[that emotion]’?”

Goal: Help participants identify more automatic thoughts in different situations and clearly understand how specific thoughts trigger specific emotions, as well as the intensity of those emotions.

3. Initiating Cognitive Restructuring

Guidance: Now that we can identify some thoughts and the emotions they trigger, today we will begin to learn how to evaluate the accuracy and usefulness of these thoughts. When you think ‘[a negative thought identified by the participant]’, let's look for evidence that supports this thought. Then, let's also consider if there is any evidence that does not support this thought, or if there are other more reasonable explanations? We can analyze this ‘evidence’ like detectives.”

Goal: Guide participants to begin objectively evaluating the evidence for their negative thoughts and to initially recognize that thoughts may not be entirely true or the only explanation.

4. Continuing Cognitive Restructuring and Introducing Behavioral Activation

Guidance: Last time, we started practicing challenging negative thoughts. Today, we will continue to explore other thoughts that trouble you and learn a new technique called behavioral activation. Sometimes, when we feel down, we tend to reduce our activities, but this can actually worsen negative emotions. Behavioral activation involves consciously scheduling and participating in activities that are likely to bring positive experiences, a sense of accomplishment, or enjoyment. Have you stopped doing anything you used to enjoy recently because you haven't been feeling well? Or is there something small you think might make you feel a little better that you would be willing to try?”

Goal: Continue to guide participants in challenging negative thoughts and introduce them to the concept of behavioral activation and its principles for improving mood, encouraging them to think about and plan to engage in positive activities.

5. Practicing Behavioral Activation and Introducing Problem-Solving

Guidance: Have you had a chance to try any of the activities we discussed last time?

How did it feel? Today, we will introduce another important CBT technique – problem-solving. It's normal to encounter various problems in life, and learning a systematic approach to analyze and solve them can help us cope with challenges better and reduce negative emotions. Do you have any specific problem that has been bothering you recently? We can go through some steps to analyze it together.”

Goal: Encourage participants to reflect on and experience the effects of behavioral activation, and introduce them to the steps of systematic problem-solving (e.g., defining the problem, brainstorming, evaluating solutions).

6. Practicing Problem-Solving and Introducing Emotion Regulation

Guidance: Last time, we started analyzing the [specific problem] you mentioned. Today, we will continue to explore possible solutions and evaluate their pros and cons. Next, we will begin to learn about emotion regulation. Emotions are a part of life, and understanding and managing our emotions is very important for mental health. When you experience strong emotions, what do you usually do? Are there any healthier ways that might help you cope with these emotions more effectively?”

Goal: Guide participants to practice problem-solving skills and introduce them to the concept of emotion regulation and some basic strategies (e.g., identifying emotions, understanding the function of emotions).

7. Practicing Emotion Regulation and Discussing Relapse Prevention

Guidance: We have learned some techniques for identifying and understanding emotions. Today, we will practice some more specific emotion regulation methods together, such as deep breathing, relaxation exercises, or active coping. In addition, we also need to prepare for the future. Thinking back to the skills we've learned over the past few weeks, what methods do you think you will use to help yourself in the future when you encounter low mood or other difficulties again? Are there any signs that might remind you that you need to pay attention to your mental health again?”

Goal: Help participants practice emotion regulation techniques and guide them to think about how to apply the learned skills to future situations and identify early warning signs of relapse.

8. Summary and Future Outlook

Guidance: This is our final interaction of the four-week intervention. Looking back, we have learned various coping skills together, such as identifying thoughts, challenging thoughts, behavioral activation, problem-solving, and emotion regulation. Which of these skills do you think have been most helpful for you? How do you plan to continue using these skills in the future to maintain your mental well-being? If you feel you need support again in the future, do you know where you can seek help?”

Goal: Summarize the entire intervention process, reinforce the skills learned by participants, encourage them to continue actively maintaining their mental health in the future, and provide information on resources for seeking further help.
