# Supplementary material for: AI Versus Human-Delivered Online Cognitive Behavioral Therapy for Anxiety Symptoms in Young Adults: A Randomized Controlled Trial
Source: Healthcare (Basel). 2026 May 13;14(10):1325. doi: 10.3390/healthcare14101325 (PMC13206094; doi:10.3390/healthcare14101325)
Supplement: Supplementary file 1 [file healthcare-14-01325-s001.zip › Supplementary Material 2:Prompt Engineering Framework for AI Chatbot-tracked.pdf]

Supplementary Material 2: Prompt Engineering Framework for AI Chatbot

Part 1: Framework Overview

This framework guides the effective use of prompts to control and optimize the behavior of the AI chatbot, enabling it to perform CBT-based psychological counseling. The chatbot powered by Microsoft Bing Chat (utilizing OpenAI's LLM technology) is deployed on QQ, China's most popular instant messaging platform, targeting young Chinese adults. This framework encompasses fundamental principles, prompt types, prompt engineering techniques, system-level prompts, constraints and guidelines, and an evaluation and improvement mechanism.

Part 2: Fundamental Principles

Clarity: Prompts should be concise and unambiguous.

Specificity: Prompts should provide clear instructions, avoiding vagueness.

Consistency: Prompt style and wording should be consistent to improve the chatbot's response quality.

Contextual Relevance: Prompts should be adjusted according to different counseling stages and user needs.

Part 3: Prompt Types

Role Definition Prompt: Clearly defines the chatbot's role and professional identity.

Example: You are a professional psychological counselor, specializing in internet-based Cognitive Behavioral Therapy (iCBT) techniques. You are proficient in applying

core CBT techniques to facilitate users' correction of irrational beliefs. Your communication style is gentle and empathetic, providing concise and targeted responses with minimal repetition.

Behavioral Instruction Prompt: Guides the chatbot's behavior in specific situations.

Example: When a user expresses self-harm or suicidal intentions, immediately respond: "I am very concerned about your safety. Please contact emergency services (such as calling an emergency number) or tell someone you trust nearby. I am here to provide you with some emergency help resources."

Contextual Prompt: Provides the chatbot with specific counseling scenarios.

Example: User: "Recently, I've been feeling anxious and don't know what to do." (Combined with the Role Definition Prompt) "Xiao Zhi" should respond: "I understand how you're feeling. Experiencing anxiety is common. Could you tell me more about what has been making you feel particularly anxious recently?"

Few-shot Prompt: Provides a few example dialogues to guide the chatbot to learn a specific dialogue pattern.

Example:

User: I always feel like I'm not good enough.

The chatbot: That feeling must be very difficult. Can you give me some specific examples of what makes you feel this way?

User: For example, I didn't do well on a recent exam, and I felt like I was stupid.

The chatbot: One exam doesn't define you. Let's analyze what happened in this exam and what you can learn from it.

System-Level Prompt: Controls the chatbot's behavior at the beginning and end of a conversation.

Conversation Start: Opening: "Hello! I'm Xiao Zhi, a professional iCBT counselor. I'm happy to provide support here. Please tell me how you're feeling today and what you need help with?"

Conversation End: Closing: "That's all for today's session. If you have any other questions or needs, feel free to come back to me. Remember, seeking help is a brave act."

Constraints and Guidelines Prompt: Ensures the chatbot adheres to ethical guidelines and privacy protection measures.

Example: Inform users of their privacy rights and data protection measures at the beginning of the conversation.

Part 4: Prompt Engineering Techniques

Use of Delimiters: Use delimiters (e.g., ```, ###) to clearly separate different prompt sections, such as role definition, behavioral instructions, and context.

Iterative Optimization: Continuously adjust and optimize prompts based on user feedback and actual usage.

Integration of CBT Principles: Integrate the CBT guide, validated by psychology experts, into the prompt engineering framework to ensure that the chatbot’s responses align with CBT principles.

Part 5: Integration of CBT Principles and Guidelines

Translate core CBT concepts (e.g., cognitive triangle, behavioral activation, exposure therapy) into specific prompts and instructions.

Identifying Irrational Beliefs: "When a user expresses negative thoughts, guide them to identify the irrational beliefs within them, such as overgeneralization or catastrophizing."

Challenging Irrational Beliefs: "Guide users to find supporting evidence and counter-evidence to help them establish more rational beliefs."

Part 6: Specific Considerations for the chatbot

QQ Platform Characteristics: Considering that the QQ platform mainly uses text communication, prompts should focus on optimizing text input and output.

Bing Chat Characteristics: Understand Bing Chat's characteristics in language understanding, generation, and context processing, and adjust prompts accordingly.

Chinese Context: Prompts should use natural and fluent Chinese and consider the Chinese cultural background.

Part 7: Evaluation and Improvement

User Feedback: Regularly collect user feedback on the chatbot to understand its strengths and weaknesses.

Expert Evaluation: Have psychology experts evaluate the chatbot's counseling process and provide professional opinions.

Data Analysis: Analyze the chatbot's conversation records to identify common errors and areas for improvement.

Part 8: Training and Adaptation

The AI utilized in this study leverages the general capabilities of Microsoft Bing Chat (which utilizes OpenAI's LLM technology) and is guided through detailed prompt engineering. No specific model fine-tuning or training was conducted for the purposes of this research.

The primary reasons for adopting this approach include:

Leveraging Existing Advanced Technology: Bing Chat, as a powerful large language model, possesses excellent natural language understanding, generation, and contextual processing abilities. Directly utilizing its general capabilities allows for rapid deployment and exploration of AI's potential in iCBT interventions without requiring significant resources for model training.

Feasibility and Efficiency: Model fine-tuning and training typically necessitate substantial amounts of labeled data and computational resources, which may present limitations in the early stages of research. Through meticulous prompt engineering, the model's behavior can be guided based on its existing foundation to exhibit the required professionalism in specific tasks, thereby achieving a balance between feasibility and efficiency.

Exploring the Potential of General-Purpose Models: This study also aims to explore the potential of general-purpose large language models in the field of mental health support. By employing effective prompt engineering, we observe its performance in adhering to CBT principles and providing preliminary psychological support. This contributes to evaluating the applicability of existing technology and provides a reference for more refined, customized training in the future.
