# Supplementary material for: AI Versus Human-Delivered Online Cognitive Behavioral Therapy for Anxiety Symptoms in Young Adults: A Randomized Controlled Trial
Source: Healthcare (Basel). 2026 May 13;14(10):1325. doi: 10.3390/healthcare14101325 (PMC13206094; doi:10.3390/healthcare14101325)
Supplement: Supplementary file 1 [file healthcare-14-01325-s001.zip › Supplementary Material 3:Peer Counselor Training and Supervision Manual-tracked.pdf]

Supplementary Material 3: Peer Counselor Training and Supervision Manual

Part 1: Introduction

Definition and Principles of Peer Counseling: Peer counseling is a process where specially trained peers use specific knowledge and skills to provide emotional support, psychological guidance, and behavioral direction to other peers in an equal and mutual manner. Its core principles include equality, confidentiality, supportiveness, non-judgmental attitude, and voluntariness.

Roles and Responsibilities of Peer Counselors: Peer counselors are listeners, supporters, guides, and resource linkers. Their main responsibilities include the following: providing active listening and emotional support; helping help-seekers clarify problems and explore solutions; guiding help-seekers to recognize their own resources and strengths; conducting crisis intervention and referrals when necessary; and adhering to ethical guidelines and receiving supervision.

Advantages and Limitations of Peer Counseling: Advantages: Easy access, reduced stigma, and promotion of understanding and support among peers. Limitations: Relatively insufficient professionalism, inability to handle severe mental illnesses, and the need for strict supervision and ethical guidelines.

Part 2: Counseling Skills

Active Listening Skills: Focusing, responding, paraphrasing, and reflecting feelings.

Effective Communication Skills: Clear expression, non-verbal communication, and empathy.

Questioning Skills: Open-ended questions, closed-ended questions, and clarifying questions.

Crisis Intervention Skills (for Depressive Tendencies): Identifying high-risk cases (focus on suicidal ideation, plans, or attempts); crisis management procedures (immediately seeking professional help); and referral resources (understanding local mental health service resources).

Part 3: Ethics and Confidentiality

Confidentiality Principles and Exceptions: Strictly adhere to confidentiality principles to protect the privacy of help-seekers. Exceptions include help-seekers with self-harm or harm-to-others tendencies, legal issues, etc. In exceptional circumstances, peer counselors should promptly seek guidance from supervisors.

Informed Consent: Before starting counseling, help-seekers should be informed of the purpose, nature, confidentiality principles, etc., of the counseling, and their consent should be obtained.

Handling Ethical Dilemmas: When encountering ethical dilemmas, peer counselors should promptly seek guidance from supervisors to jointly explore solutions.

Part 4: Supervision

Purpose and Importance of Supervision: Supervision aims to improve the professional competence of peer counselors, protect the rights and interests of help-seekers, and safeguard the physical and mental health of peer counselors.

Supervision Methods and Frequency: Individual supervision, group supervision, case discussions, audio/video recording supervision, etc. Regular supervision is recommended.

Content and Focus of Supervision: Case discussions, counseling skills guidance, ethical issue discussions, self-care for peer counselors, etc.

Self-Care for Peer Counselors: Pay attention to one's own emotions and stress, learn self-regulation, and seek support.

Part 5: Assessment

Formative Assessment: Attendance rate, online course progress.

Summative Assessment: Case report, evaluated by two doctoral-level supervisors based on:

Theoretical Application: Correct application of learned theories in case analysis.

Skills Application: Appropriate use of counseling skills.

Ethical Awareness: Adherence to ethical guidelines.

Depth of Analysis: Depth and comprehensiveness of the case analysis.

Report Writing: Clarity and fluency of the report.

Part 6: Supervision Process

Supervision Team: Composed of two professionals with doctoral degrees in psychology or related fields.

Supervision Format: Primarily a combination of individual and group supervision, supplemented by case discussions and audio/video recording supervision as needed.

Supervision Frequency: In principle, once a week, 1-2 hours each time. Adjustments can be made based on the peer counselors' needs.

Supervision Content:

Case Supervision: In-depth analysis and discussion of cases handled by peer counselors to help them improve their counseling skills and problem-solving abilities.

Ethical Supervision: Discussion of ethical issues encountered during counseling, providing ethical guidance.

Personal Growth Supervision: Focus on the peer counselors' personal growth and emotional state, helping them better cope with work stress.
