# Supplementary material for: AI Versus Human-Delivered Online Cognitive Behavioral Therapy for Anxiety Symptoms in Young Adults: A Randomized Controlled Trial
Source: Healthcare (Basel). 2026 May 13;14(10):1325. doi: 10.3390/healthcare14101325 (PMC13206094; doi:10.3390/healthcare14101325)
Supplement: Supplementary file 1 [file healthcare-14-01325-s001.zip › Supplementary Material 4:Semi-structured Interview Guide-tracked.pdf]

Supplementary Material 4: Semi-structured Interview Guide

I. Interview Introduction

Hello, thank you for participating in this interview. This interview aims to understand your experiences and perspectives on using psychological counseling, including your experiences with different counseling modalities. The interview will take approximately 15–20 minutes. For data analysis purposes, we will be audio-recording the interview. Please be assured that all audio data will be anonymized and used for academic research purposes only. Your personal information will not be disclosed. You are welcome to ask any questions you may have at any time.

II. Interview Themes and Questions

Part 1: In-Process Recall (IPR)

1. Please recall the entire counseling process and describe your psychological feelings and mental activities at different stages (e.g., beginning, middle, end).
2. During the counseling process, were there any specific moments that stood out to you? Please describe your thoughts and feelings at those times.
3. During the counseling process, did you experience any questions, confusions, or hesitations? If so, what were they?
4. What aspects of the counseling process did you find most helpful? What aspects, if any, could be improved?

Part 2: Comparative Aspects of AI and Human Counseling

1. What are your perceptions of the differences between receiving counseling from an AI and from a human counselor? Please consider aspects such as

communication style, the perceived presence of empathy, use of language, and any other relevant factors.

2. From your perspective, what are the relative advantages and disadvantages of AI-mediated versus human-delivered counseling?
3. During your interactions with the AI counselor, to what extent did you feel you were communicating with a machine versus a human? Please elaborate on your reasoning. How accurate did you perceive the AI's comprehension of your communication to be?

Part 3: Attitudes towards AI in Counseling

1. What are your general views on the application of AI in the field of psychological counseling? What potential benefits and risks do you associate with this application?
2. What are your views on the potential role of AI in intimate human relationships (e.g., romantic partnerships, close friendships)?
3. In your opinion, what should be the future direction of AI development within psychological counseling?
4. Would you consider using AI for psychological counseling again in the future? Please explain your reasoning.
5. If you were to rate the overall effectiveness of the AI in addressing your concerns on a scale of 0 (not at all effective) to 10 (completely effective), what score would you give? Please explain your rating.

6. What recommendations would you offer for improving the effectiveness and user experience of AI-mediated counseling?

III. Interview Closing

This concludes the interview. Thank you for your participation and valuable time. Your feedback is highly valuable for us to improve psychological counseling services, especially the application of AI in this field. We wish you a pleasant day.
